# Repetitive Transcranial Magnetic Stimulation and Tai Chi Chuan for Older Adults With Sleep Disorders and Mild Cognitive Impairment: A Randomized Clinical Trial

**DOI:** 10.1001/jamanetworkopen.2024.54307

**Published:** 2025-01-10

**Authors:** Zhizhen Liu, Lin Zhang, Linxin Bai, Zhenxing Guo, Jiahui Gao, Yongsheng Lin, Yongjin Zhou, Jinghui Lai, Jing Tao, Lidian Chen

**Affiliations:** 1 National-Local Joint Engineering Research Center of Rehabilitation Medicine Technology, Fujian University of Traditional Chinese Medicine, Fuzhou, Fujian, China; 2 College of Rehabilitation Medicine, Fujian University of Traditional Chinese Medicine, Fuzhou, Fujian, China; 3 School of Biomedical Engineering, Medical School, Shenzhen University, Shenzhen, Guangdong, China; 4 Marshall Laboratory of Biomedical Engineering, Shenzhen University, Shenzhen, Guangdong, China; 5 The Affiliated Rehabilitation Hospital, Fujian University of Traditional Chinese Medicine, Fuzhou, Fujian, China; 6 Fujian Key Laboratory of Cognitive Rehabilitation, Fuzhou, Fujian, China

## Abstract

**Importance:**

Sleep disorders and mild cognitive impairment (MCI) commonly coexist in older adults, increasing their risk of developing dementia. Long-term tai chi chuan has been proven to improve sleep quality in older adults. However, their adherence to extended training regimens can be challenging. Repetitive transcranial magnetic stimulation (rTMS) is a neuromodulation technique that may enhance the benefits of exercise.

**Objective:**

To investigate whether 1-Hz rTMS of the right dorsolateral prefrontal cortex could enhance the clinical benefits of tai chi chuan in improving sleep quality and cognitive function among older adults with sleep disorders and MCI.

**Design, Setting, and Participants:**

This 2-arm, sham-controlled, assessor-masked randomized clinical trial was conducted at a university hospital in China between October 2022 and February 2024. Adults aged 60 to 75 years with sleep disorders and MCI were eligible. Data analysis was performed from February to May 2024.

**Intervention:**

Participants were randomized in a 1:1 ratio to an experimental group (tai chi chuan and 1-Hz rTMS) or a sham group (tai chi chuan and sham rTMS). Each participant received 30 sessions of personalized rTMS targeting the right dorsolateral prefrontal cortex, and the sham group underwent the same procedure. The 2 groups received 30 sessions of 60 minutes of the 24-form simplified tai chi chuan, 5 times per week for 6 weeks.

**Main Outcomes and Measures:**

The primary outcomes were subjective sleep quality assessed by the Pittsburgh Sleep Quality Index (PSQI), in which scores range from 0 to 21, with lower scores indicating a healthier sleep quality, and global cognitive function assessed by the Montreal Cognitive Assessment (MoCA), in which scores range from 0 to 30, with higher scores indicating less cognitive impairment. The secondary outcomes included measures of objective sleep actigraphy, anxiety and depression scales, and other cognitive subdomains. Assessments were performed at baseline, 6 weeks after the intervention, and at the 12-week follow-up.

**Results:**

A total of 110 participants (mean [SD] age, 67.9 [4.6] years; 68 female [61.8%]) were randomized to the experimental group (n = 55) and the sham group (n = 55) and included in the intention-to-treat analysis. At 6 weeks after the intervention, compared with the sham group, the experimental group showed a lower PSQI score (between-group mean difference, −3.1 [95% CI, −4.2 to −2.1]; *P* < .001) and a higher MoCA score (between-group mean difference, 1.4 [95% CI, 0.7-2.1]; *P* < .001). The per-protocol dataset analyses and 12-week follow-up showed similar results. The generalized estimated equation model revealed an interaction effect between the PSQI score (mean difference, −2.1 [95% CI, −3.1 to −0.1]; *P* < .001) and the MoCA total score (mean difference, 0.9 [95% CI, 0.1-1.6]; *P* = .01). There were 7 nonserious, unrelated adverse events (experimental group: 2; sham group: 5) with no significant difference between the 2 groups.

**Conclusions and Relevance:**

In this randomized clinical trial, the findings suggest that 1-Hz rTMS enhanced the clinical benefits of tai chi chuan in improving sleep quality and cognitive function among older adults with sleep disorders and MCI, which may be related to alterations in neural plasticity. These findings provide novel data on nonpharmacologic strategies for the rehabilitation of sleep disorders and may delay or even prevent MCI.

**Trial Registration:**

Chinese Clinical Trial Registry Identifier: ChiCTR2200063274

## Key Points

**Question:**

Can repetitive transcranial magnetic stimulation (rTMS) of the right dorsolateral prefrontal cortex enhance the clinical benefits of tai chi chuan in older adults with sleep disorder and mild cognitive impairment?

**Findings:**

In this randomized clinical trial of 110 older adults in China, 1-Hz rTMS targeting the right dorsolateral prefrontal cortex improved the benefits of tai chi chuan for sleep and cognitive performance in older adults compared with sham rTMS at the 6-week intervention and the 12-week follow-up.

**Meaning:**

The findings of this study provide data on nonpharmacologic strategies for the rehabilitation of sleep disorders and may delay or prevent mild cognitive impairment among older adults.

## Teaser

This randomized clinical trial assesses whether repetitive transcranial magnetic stimulation (rTMS) of the right dorsolateral prefrontal cortex enhances the clinical benefits of tai chi chuan in older adults in China with sleep disorder and mild cognitive impairment (MCI).

## Introduction

Sleep disorders are a considerable global health concern, and nearly half of adults over 60 years of age experience sleep disorders.^1,2^ As people age, sleep disorders can exacerbate cognitive decline and are recognized as independent risk factors for mild cognitive impairment (MCI),^3^ along with a 30% increased risk of progressing dementia.^4^ Sleep homeostasis maintains the fundamental physiologic health of neurons by clearing potentially harmful metabolic waste products, which promotes the consolidation and integration of memories.^5^ Therefore, early improvement in sleep quality may be crucial for preventing cognitive decline in older adults.^6,7^ As pharmacologic approaches have shown unsatisfactory results,^8^ the American Geriatrics Society recommends prioritizing nonpharmacologic strategies to improve sleep issues in older adults.^9^

Several studies have revealed that exercise can improve sleep quality.^10,11,12^ Tai chi chuan, as a mind-body exercise, has been shown to alleviate sleep disorder symptoms^13^ and delay the progression of cognitive impairment in older adults,^14^ with its benefits linked to activation of the prefrontal cortex (PFC).^15^ However, the functional gains of tai chi chuan remain limited,^16^ and adhering to a long-term exercise regime is challenging for older adults.

The dorsolateral PFC (DLPFC) serves as a central regulator of cognitive function.^17,18^ Sleep disorders can disrupt the balance of excitability and inhibitory activity in the brain, including the DLPFC,^19^ which contributes to cognitive decline. The DLPFC is commonly targeted in repetitive transcranial magnetic stimulation (rTMS) treatment for sleep disorders.^20^ By targeting the DLPFC,^21^ low-frequency rTMS can promote cortical network and subcortical plasticity changes,^22^ effectively improving sleep quality.^23,24^ Tai chi chuan can induce widespread changes in brain plasticity,^25^ and when combined with rTMS, it can lead to more lasting alterations in neural plasticity.^26,27^ These alterations enhance brain benefits through tai chi chuan, such as significantly improving sleep quality among community-dwelling older adults, as found in small sample trials.^28^ However, no large-scale randomized clinical trials have reported whether rTMS could enhance the clinical benefit of tai chi chuan for improving sleep quality and cognitive function in older adults with sleep disorders and MCI, and its efficacy remains unclear.

In this study, we conducted a double-blind, sham-controlled, 2-arm randomized clinical trial to examine whether 1-Hz rTMS targeting the right DLPFC would be superior to sham rTMS in augmenting the benefits of tai chi chuan on sleep quality and cognitive function among older adults with sleep disorders and MCI. We assessed sleep quality and cognitive function at the end of a 6-week intervention and at a 12-week follow-up. We hypothesized that 1-Hz rTMS would be superior to sham rTMS in enhancing the clinical benefits of tai chi chuan.

## Methods

### Study Design

The trial protocol and statistical analysis plan are available in Supplement 1. The ethics committees of The Affiliated Rehabilitation Hospital, Fujian University of Traditional Chinese Medicine, approved the study. All participants provided written informed consent. The study followed the Consolidated Standards of Reporting Trials (CONSORT) reporting guideline.

### Participants

This study was a 2-arm, sham-controlled, assessor-masked randomized clinical trial conducted at a university hospital in China between October 2022 and February 2024. Participants were recruited at community centers. Inclusion criteria were having a clinical diagnosis of sleep disorders, experiencing MCI without dementia, being aged 60 to 75 years, engaging in no regular practice of moderate-intensity exercise or tai chi chuan (>3 times per week, >20 minutes per session) in the past 3 months, and providing informed consent and participating voluntarily. Exclusion criteria included a Geriatric Depression Scale-15 score of 9 or more, in which scores range from 0 to 15, with higher scores indicating severe depression, or any antipsychotics use in the past month; major confounding conditions that induce sleep disorders, such as severe chronic diseases or pain disorders; cognitive impairment from other causes; any physical disability that precluded completing tai chi chuan and rTMS; and regular noninvasive stimulation and hypnotic drug therapy within 2 weeks before enrollment.

### Randomization and Masking

Randomization was conducted by independent researchers via an automated permuted block algorithm with a block size of 4. The study participants were randomly assigned to the experimental group (tai chi chuan and 1-Hz rTMS) or the sham group (tai chi chuan and sham rTMS) at a 1:1 ratio (Figure). The outcome evaluators and statistical analysts were masked from group allocation. The tai chi chuan coach was unaware of the group allocation and the nature of the intervention.

**Figure.**
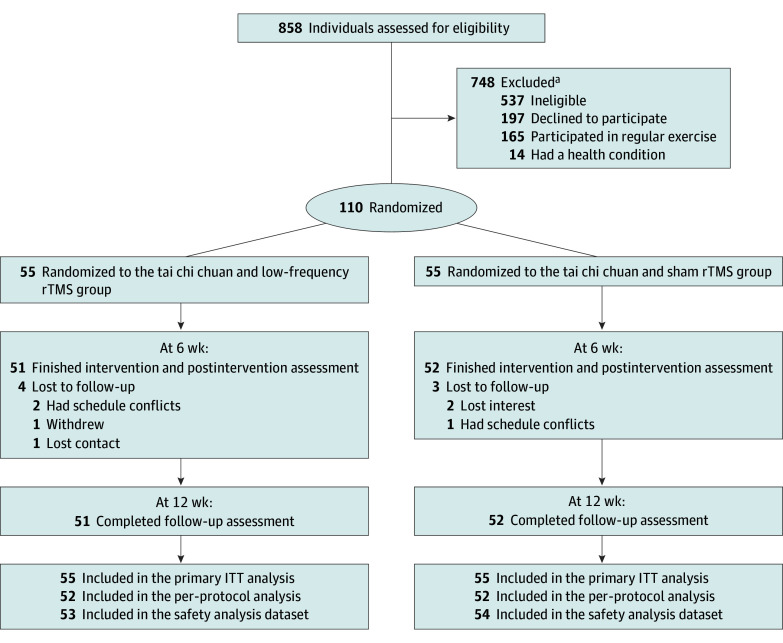
Study Flow Diagram ITT indicates intention-to-treat; rTMS, repetitive transcranial magnetic stimulation. ^a^Participants could be excluded for more than 1 reason.

### Intervention

#### Tai Chi Chuan

The tai chi chuan training sessions were conducted every morning for 6 weeks, with 5 sessions per week, each lasting 60 minutes. Tai chi chuan is a mind-body exercise that integrates the musculoskeletal, sensory, and cognitive systems. We adopted the Yang-style, 24-form tai chi chuan training plan, which is the most common and studied form or style in the literature. The tai chi chuan training was instructed by certified instructors with at least 5 years of experience directing interventions. The detailed course arrangements are provided in eTables 1 and 2 in Supplement 2.

#### rTMS

rTMS was administered via a Rapid^2^ stimulator (Magstim), equipped with a standard 70-mm figure-8 coil. All participants underwent a 20-minute session of active or sham rTMS, administered 5 times per week for 6 weeks in the evening. The resting motor threshold was determined by measuring the muscle response of the abductor pollicis brevis and was set at 80%.^29^ In accordance with current guidelines, 1-Hz rTMS was applied over the right DLPFC.^20^ The precise rTMS location and continuous monitoring relative to the right DLPFC were achieved using a neuronavigation system. In the experimental group, each session of rTMS consisted of a sequence of 60 stimulation pulses per string, with a string interval of 1 second for a total of 60 strings, which amounted to 1200 stimulation pulses over a continuous duration of 20 minutes. The sham group received the same rTMS intervention using a sham coil, which had the same symmetrical mechanical design. The rTMS intervention was administered by qualified rehabilitation therapists. Further details are provided in the trial protocol and statistical analysis plan in Supplement 1.

### Sample Size Estimation

Given the absence of studies on the combined intervention of tai chi chuan and rTMS, the sample size calculation was conducted by referring to studies demonstrating improvements in Pittsburgh Sleep Quality Index (PSQI) scores as a result of rTMS^30^ or tai chi chuan interventions.^31^ The required sample size for the PSQI group was calculated to be 31 participants and for the tai chi chuan intervention, 49 participants. G*Power, version 3.1 (Heinrich-Heine-Universität Düsseldorf), considering a 10% dropout rate, was used to provide 80% power to achieve statistical significance at the 5% 2-sided level for comparisons between the 2 intervention groups.

### Outcome Measures

All participants were assessed at baseline, after the 6-week intervention, and at the 12-week follow-up. A standardized implementation plan and standard operating procedures were followed.

The primary outcomes included the PSQI for assessing sleep quality,^32^ in which scores range from 0 to 21, with lower scores indicating a healthier sleep quality, and the Montreal Cognitive Assessment (MoCA) for assessing global cognition, in which scores range from 0 to 30, with higher scores indicating less cognitive impairment.^33^ The secondary outcomes for cognitive function assessment included the Wechsler Scale–Revised memory quotient, in which scores range from 40 to 160, with higher scores indicating better memory performance, and the Victoria Stroop test (conditions 1-3) and the Trail Making Test Part B, in which scores are measured by the time required to complete a task. The Hamilton Anxiety Rating Scale, in which scores range from 0 to 56, with higher scores indicating more severe anxiety symptoms; the Hamilton Depression Rating Scale, in which scores range from 0 to 52, with higher scores indicating more severe depressive symptoms; and the 36-Item Short Form Health Survey, in which scores range from 43 to 150 for each subscale, with higher scores indicating better health status or quality of life, were used to assess anxiety and depression levels, as well as quality of life. Sleep quality was evaluated with the Epworth Sleepiness Scale, in which scores range from 0 to 24, with higher scores indicating greater levels of daytime sleepiness, and with actigraphy. The participants were instructed to wear a wrist actigraph on their nondominant wrist for 24 hours over the course of 7 days.^34^ Objective sleep data were collected from the returned wrist actigraph, which objectively measured the 7-day mean of sleep parameters, including sleep efficiency, wake time after sleep onset, the number of awakenings per night, sleep-onset latency, total sleep time, and median wake time per awakening. Importantly, for clarity, wrist actigraph data were collected only at baseline and after the intervention. Additionally, the dosage and frequency of any hypnotic medication were recorded.

### Statistical Analysis

Data analysis was performed from February to May 2024. All participants who provided informed consent were included in the intention-to-treat (ITT) analysis. The per-protocol set included participants who adhered to the treatment protocol. The safety analysis dataset included all participants who received at least 1 treatment and underwent a safety assessment after randomization. Multiple imputation was used for missing data on primary outcome measures at baseline, after the intervention, and at follow-up. The intervention effects on primary outcome measures (PSQI and MoCA scores) were primarily assessed using the ITT analysis, and the per-protocol set was used for sensitivity analysis. Secondary outcome measures were assessed mainly via the per-protocol set. Analysis was conducted using the generalized estimated equation model, with group and time as the main effects and baseline measurements and the International Physical Activity Questionnaire score as covariates (eTable 3 in Supplement 2). Continuous variables were reported as mean (SD) or median (IQR). At individual time points (ie, baseline, after the intervention, and at follow-up), generalized linear models were used for between-group comparisons, with baseline as a covariate, similarly applied to objective sleep parameters. Categorical variables were presented as counts and percentages and were analyzed using the χ^2^ test or the Fisher exact test. The subgroup analysis was conducted based on sex, age, educational level, body mass index, and the Geriatric Depression Scale-15 score. All statistical analyses were conducted using SPSS, version 24.0 (IBM Inc). Two-sided *P* < .02 for the primary outcome measures and 2-sided *P* < .05 for the secondary outcome measures indicated statistical significance. Further details can be found in the trial protocol and statistical analysis plan in Supplement 1.

## Results

### Participant Characteristics

Among 858 participants screened for eligibility, a total of 110 participants (mean [SD] age, 67.9 [4.6] years; 68 female [61.8%] and 42 male [38.2%]) were randomly assigned to the experimental group (n = 55) or the sham group (n = 55) after baseline assessment and were enrolled, randomized, and included in the ITT analysis. Of the total participants, 103 (93.6%) completed the 6-week intervention and 12-week follow-up evaluation. All of the participants were included in the ITT analysis, and those who completed the 12-week evaluation were included in the per-protocol analysis. A total of 7 adverse events occurred in the 2 groups and were included in the safety analysis. As shown in the Figure, during the 6-week intervention period, all 103 patients completed rTMS treatment. There was no significant difference in the compliance rate, as measured by tai chi chuan class attendance, between the experimental group (92.4%) and the sham group (93.7%). The baseline demographic and clinical characteristics of the participants are summarized in Table 1.

**Table 1.** Baseline Characteristics of Participants^a^

Characteristic	Study group (N = 110)	
	Experimental (n = 55)	Sham (n = 55)
Age, mean (SD), y	67.8 (4.5)	68.0 (4.6)
Sex		
Female	37 (67.3)	31 (56.4)
Male	18 (32.7)	24 (43.6)
Education, mean (SD), y	11.0 (2.6)	11.2 (2.7)
BMI, mean (SD)	23.5 (0.4)	23.8 (0.4)
GDS-15, median (IQR)	2.0 (1.0-4.0)	2.0 (1.0-4.0)
Diabetes		
No	42 (76.4)	45 (81.8)
Yes	13 (23.6)	10 (18.2)
Hypertension		
No	34 (61.8)	32 (58.2)
Yes	21 (38.2)	23 (41.8)
Medication use in the last 2 wk^b^		
No	26 (47.3)	24 (43.6)
Yes	29 (52.7)	31 (56.4)
Smoking		
Never	54 (98.2)	48 (87.3)
Current	1 (1.8)	7 (12.7)
Alcohol consumption		
Never	52 (94.5)	51 (92.7)
Current	3 (5.5)	4 (7.3)
Drinking tea (ie, caffeine consumption)		
No	42 (76.4)	38 (69.1)
Yes	13 (23.6)	17 (30.9)

Abbreviations: BMI, body mass index (calculated as weight in kilograms divided by height in meters squared); GDS-15, Geriatric Depression Scale-15 (in which scores range from 0 to 15, with higher scores indicating severe depression).

^a^ Data are presented as the No. (%) of participants unless otherwise indicated.

^b^ The use of drugs for controlling conditions such as diabetes and hypertension.

### Primary Outcomes

Compared with those of the sham group, the PSQI scores of the experimental group decreased after the intervention (mean difference, −3.1 [95% CI, −4.2 to −2.1]; *P* < .001) and at follow-up (mean difference, −2.1 [95% CI, −3.1 to −0.1]; *P* < .001). Furthermore, the experimental group manifested improvements in MoCA scores after the intervention (mean difference, 1.4 [95% CI, 0.7-2.1]; *P* < .001) and at follow-up (mean difference, 0.9 [95% CI, 0.1-1.6]; *P* = .01). An interaction effect between the PSQI and the MoCA was observed, as indicated by the generalized estimated equation analysis. Similar findings were also observed in the per-protocol analysis (Table 2).

**Table 2.** Primary Outcome of Generalized Estimated Equation Analysis^a^

Measurement	Study group, mean (SD)		Mean difference (95% CI)	*P* value		
	Experimental	Sham		Group effect	Time effect	Interaction effect
**PSQI**						
ITT						
No. of participants	55	55	NA	NA	NA	NA
Baseline	12.8 (2.8)	12.5 (2.7)	0.3 (−0.7 to 1.4)	<.001	<.001	<.001
6 wk	6.7 (3.3)	9.5 (3.6)	−3.1 (−4.2 to −2.1)^b^			
12 wk	7.0 (3.0)	8.8 (3.5)	−2.1 (−3.1 to −0.1)^b^			
Per protocol						
No. of participants	51	52	NA	NA	NA	NA
Baseline	12.8 (2.8)	12.5 (2.7)	0.3 (−0.8 to 1.4)	<.001	<.001	<.001
6 wk	6.6 (3.4)	9.6 (3.6)	−3.2 (−4.3 to −2.1)^b^			
12 wk	6.8 (3.0)	8.8 (3.6)	−2.2 (−3.3 to −1.1)^b^			
**MoCA**						
ITT						
No. of participants	55	55	NA	NA	NA	NA
Baseline	23.2 (1.8)	22.6 (2.0)	0.6 (−0.1 to 1.3)	<.02	<.001	.01
6 wk	26.1 (2.0)	24.4 (2.5)	1.4 (0.7 to 2.1)^b^			
12 wk	26.2 (2.2)	24.9 (2.5)	0.9 (0.1 to 1.6)^b^			
Per protocol						
No. of participants	51	52	NA	NA	NA	NA
Baseline	23.3 (1.7)	22.6 (2.0)	0.7 (−0.9 to 1.3)	<.001	<.001	.007
6 wk	26.3 (1.9)	24.4 (2.5)	1.5 (0.8 to 2.3)^b^			
12 wk	26.3 (2.2)	24.9 (2.5)	1.4 (0.2 to 1.8)^b^			

Abbreviations: ITT, intention to treat; MoCA, Montreal Cognitive Assessment (in which scores range from 0 to 30, with higher scores indicating less cognitive impairment); NA, not applicable; PSQI, Pittsburgh Sleep Quality Index (in which scores range from 0 to 21, with lower scores indicating a healthier sleep quality).

^a^ A generalized estimated equation model, with baseline measurement as the covariate, was used to analyze the data. *P* < .02 indicates statistical significance in the group differences.

^b^ At the same time, between-group comparisons remained statistically significant after applying generalized linear models with baseline as a covariate.

### Secondary Outcomes

Compared with the sham group, the experimental group showed significant improvements in the Wechsler Scale–Revised memory quotient scores (8.8 [95% CI, 5.0-12.6]; *P* < .001) and in the 36-Item Short Form Health Survey scores (2.8 [95% CI, 0.5 to 5.1]; *P* = .02), as well as greater reductions in the Epworth Sleepiness Scale scores (−1.6 [95% CI, −2.8 to −0.4]; *P* = .01), the Hamilton Anxiety Rating Scale scores (−1.9 [95% CI, −2.9 to −0.9]; *P* < .001), and the Hamilton Depression Rating Scale scores (−1.7 [95% CI, −2.5 to −1.0]; *P* < .001). Additionally, the experimental group performed better on the Victoria Stroop test for condition 1 (−1.8 seconds [95% CI, −3.5 to −0.1 seconds]; *P* = .04), condition 2 (−1.4 seconds [95% CI, −3.4 to −0.6 seconds]; *P* = .02), and condition 3 (−5.1 seconds [95% CI, −9.8 to −0.4 seconds]; *P* = .03) after the intervention. However, there was no significant difference in the Trail Making Test Part B time between the groups after the intervention.

During the 12-week follow-up period, the experimental group showed significant improvements in the Wechsler Scale–Revised memory quotient score (7.0 [95% CI, 3.0-10.9]; *P* = .001) and used less time in the Victoria Stroop test for conditions 2 (−2.4 seconds [95% CI, −4.5 to −0.4 seconds]; *P* = .02) and 3 (−6.5 seconds [95% CI, −12.0 to −1.2 seconds]; *P* = .02) and in the Trail Making Test Part B (−19.8 seconds [95% CI, −34.5 to −5.2 seconds]; *P* = .008). Furthermore, lower Hamilton Depression Rating Scale scores (−1.3 [95% CI, −2.0 to −0.5]; *P* = .001) were recorded. Nonetheless, there were no significant differences in other indicators between the groups at follow-up (Table 3).

**Table 3.** Secondary Outcome of Generalized Estimated Equation Analysis^a^

Measurement	Study group, mean (SD) (N = 103)		Mean difference (95% CI)	*P* value		
	Experimental (n = 51)	Sham (n = 52)		Group effect	Time effect	Interaction effect
**Wechsler Scale–Revised memory quotient** ^b^						
Baseline	99.3 (13.3)	98.0 (12.9)	1.3 (−3.9 to 6.4)	<.001	<.001	<.001
6 wk	114.0 (14.0)	104.4 (11.7)	8.8 (5.0 to 12.6)^c^			
12 wk	112.0 (13.1)	104.3 (13.6)	7.0 (3.0 to 10.9)^c^			
**Epworth Sleepiness Scale** ^d^						
Baseline	7.0 (4.9)	5.8 (4.0)	1.2 (−0.5 to 3.0)	.04	<.001	.02
6 wk	3.5 (3.2)	4.8 (3.6)	−1.6 (−2.8 to −0.4)^c^			
12 wk	3.8 (3.3)	4.1 (3.6)	−0.6 (−1.9 to 0.7)			
**Victoria Stroop test condition 1, seconds** ^e^						
Baseline	18.2 (5.9)	19.0 (5.8)	−0.8 (−3.1 to 1.5)	.08	.95	.29
6 wk	17.5 (5.4)	19.9 (6.3)	−1.8 (−3.5 to −0.1)^c^			
12 wk	17.8 (7.4)	19.8 (6.2)	−1.5 (−3.7 to 0.7)			
**Victoria Stroop test condition 2, seconds** ^e^						
Baseline	22.5 (6.1)	25.1 (6.4)	−2.6 (−5.1 to −0.2)	.07	.002	.59
6 wk	20.8 (5.5)	23.5 (6.7)	−1.4 (−3.4 to −0.6)^c^			
12 wk	19.9 (5.3)	23.5 (6.6)	−2.4 (−4.5 to −0.4)^c^			
**Victoria Stroop test condition 3, seconds** ^e^						
Baseline	38.4 (13.3)	43.4 (14.2)	−5.0 (−10.4 to 0.4)	.09	.01	.80
6 wk	35.2 (13.8)	40.3 (10.7)	−5.1 (−9.8 to −0.4)^c^			
12 wk	33.7 (13.1)	40.2 (14.5)	−6.5 (−12.0 to −1.2)^c^			
**Trail Making Test Part B, seconds** ^e^						
Baseline	157.2 (54.7)	156.3 (44.8)	0.9 (−18.7 to 20.4)	.006	.27	.07
6 wk	150.0 (51.0)	165.8 (46.5)	−16.2 (−32.5 to 0)			
12 wk	141.4 (42.4)	160.9 (46.4)	−19.8 (−34.5 to −5.2)^c^			
**Hamilton Anxiety Rating Scale** ^f^						
Baseline	7.9 (3.2)	7.5 (3.4)	0.4 (−0.8 to 1.7)	.003	<.001	.003
6 wk	3.7 (3.0)	5.5 (2.8)	−1.9 (−2.9 to −0.9)^c^			
12 wk	3.5 (2.6)	4.1 (2.5)	−0.7 (−1.6 to 0.2)			
**Hamilton Depression Rating Scale** ^g^						
Baseline	5.6 (3.1)	5.4 (2.5)	0.2 (−0.8 to 1.3)	<.001	<.001	.004
6 wk	2.1 (1.7)	3.7 (2.3)	−1.7 (−2.5 to −1.0)^c^			
12 wk	1.9 (1.9)	3.1 (2.0)	−1.3 (−2.0 to −0.5)^c^			
**36-Item Short Form Health Survey** ^h^						
Baseline	104.0 (7.6)	104.1 (7.0)	0.1 (−2.7 to 2.8)	.01	.07	.09
6 wk	106.1 (5.7)	103.3 (6.9)	2.8 (0.5 to 5.1)^c^			
12 wk	103.4 (16.5)	95.5 (28.7)	7.9 (−1.1 to 16.8)			

^a^ A generalized estimated equation model, with baseline measurement as the covariate, was used to analyze the data.

^b^ Scores range from 40 to 160, with higher scores indicating better memory performance.

^c^ At the same time, between-group comparisons remained statistically significant after applying generalized linear models with baseline as a covariate.

^d^ Scores range from 0 to 24, with higher scores indicating greater levels of daytime sleepiness.

^e^ Scores assess cognitive function by measuring the time required to complete a task.

^f^ Scores range from 0 to 56, with higher scores indicating more severe anxiety symptoms.

^g^ Scores range from 0 to 52, with higher scores indicating more severe depressive symptoms.

^h^ Scores range from 43 to 150 for each subscale, with higher scores indicating better health status or quality of life.

Interaction effects were observed for the Wechsler Scale–Revised memory quotient, the Epworth Sleepiness Scale, the Hamilton Anxiety Rating Scale, and the Hamilton Depression Rating Scale scores. In the Victoria Stroop test, there were no interaction effects among the 3 conditions, but there were time effects for conditions 2 and 3. There were no interaction effects for the Trail Making Test Part B or the 36-Item Short Form Health Survey, but there were group effects (Table 3).

Among objective sleep parameters, compared with the sham group, the experimental group manifested increased sleep efficiency after the intervention (mean difference, 3.0 [95% CI, 0.7-5.3]; *P* = .01). However, there were intergroup differences in sleep latency both before and after the intervention (Table 4).

**Table 4.** Objective Sleep Parameters of Generalized Linear Models Analysis^a^

Measurement	Study group (N = 103)		Mean difference (95% CI)	*P* value
	Experimental (n = 51)	Sham (n = 52)		
**Sleep efficiency, mean (SD), %**				
Baseline	82.8 (5.8)	80.6 (6.6)	2.2 (−0.2 to 4.6)	.09
6 wk	83.8 (4.7)	80.8 (7.2)	3.0 (0.7 to 5.3)	.01
**Sleep latency, min**				
Baseline	38.8 (28.5 to 42.8)	41.6 (32.0 to 51.5)	−6.8 (−13.9 to 0.2)	.03
6 wk	37.0 (25.0 to 42.5)	42.5 (28.4 to 57.4)	−6.2 (−13.9 to 1.5)	.03
**No. of awakenings after falling asleep**				
Baseline	1.9 (1.5 to 2.1)	1.9 (1.7 to 2.2)	−0.2 (−0.4 to 0)	.19
6 wk	1.8 (1.4 to 2.0)	1.8 (1.7 to 2.1)	−0.1 (−0.2 to 0.1)	.32
**Mean awaken time, min**				
Baseline	21.4 (13.8 to 25.7)	20.3 (15.4 to 21.8)	2.6 (−4.8 to 3.1)	.93
6 wk	18.9 (12.1 to 22.3)	18.9 (14.0 to 22.9)	−0.5 (−3.6 to 2.6)	.92
**Wake time after sleep, min**				
Baseline	183.0 (164.4 to 199.4)	183.0 (152.3 to 209.7)	2.8 (−13.1 to 18.8)	.97
6 wk	188.6 (158.3 to 207.5)	188.6 (166.5 to 203.1)	4.2 (−13.5 to 22.0)	.73
**Total sleep time, min**				
Baseline	377.8 (358.7 to 388.1)	377.8 (351.5 to 406.1)	−5.9 (−25.4 to 13.7)	.74
6 wk, mean (SD)	374.2 (38.2)	364.1 (43.5)	12.4 (−1.2 to 26.0)	.22

^a^ Generalized linear models, with baseline measurement as the covariate, were used to analyze the data. Data are presented as median (IQR) values unless otherwise indicated.

Both groups reduced their use of hypnotic medication, although no significant between-group differences were observed (eTable 4 and eFigure 1 in Supplement 2). Additionally, the 12-week subgroup analysis demonstrated that the experimental group exhibited improvements in PSQI and MoCA scores, particularly among females, individuals with a body mass index of 24 or less (calculated as weight in kilograms divided by height in meters squared), and those with more than 10 years of education (eFigures 2 and 3 in Supplement 2).

### Adverse Events

There were 7 nonserious, unrelated adverse events (experimental group: 2; sham group: 5). Two participants (3.7%) in the sham group experienced dizziness. One participant (1.9%) in the experimental group and 1 participant (1.9%) in the sham group reported falls. Additionally, 1 participant (1.9%) in the experimental group and 1 participant (1.9%) in the sham group reported hospitalization. One participant (1.9%) in the sham group required an emergency department visit. There was no statistically significant difference in adverse events between the 2 groups. Based on the assessment by a specialist using a standardized reporting system, the occurrence of adverse events was unrelated to the intervention measures of this study (eTable 5 in Supplement 2).

## Discussion

To our knowledge, this is the first study to find that 1-Hz rTMS of the right DLPFC was more effective than sham rTMS in augmenting the benefits of tai chi chuan. We found that tai chi chuan combined with 1-Hz rTMS significantly improved subjective sleep quality, as indicated by the PSQI, and global cognitive function, as measured by the MoCA. Additionally, there was an improvement in sleep efficiency, assessed by actigraphy, and a reduction in the use of hypnotic medication. Compared with the sham group, the experimental group showed significant improvements in cognitive functions such as memory function, executive function, and attention in various subdomains. The beneficial effects on sleep and cognition were still evident at the 12-week follow-up.

Notably, the enhancement in sleep efficiency of the experimental group was modest, possibly because achieving an improvement in objective sleep efficiency often requires a longer period.^12,35^ Furthermore, our research revealed that a 6-week combined intervention can improve depression and quality of life in older adults with sleep disorders and MCI.^36^ The alleviation of depression could last up to 12 weeks, possibly due to better sleep quality.^37,38^ However, there was no significant difference in anxiety and quality of life between the groups at 12 weeks, likely because participants had favorable baseline anxiety and daily functioning, resulting in only modest improvements. These findings provide evidence that 1-Hz rTMS stimulation over the right DLPFC enhances the clinical benefits of tai chi chuan in improving sleep quality and cognitive function in older adults with sleep disorders and MCI.

The potential mechanism supporting the current findings may be the complementary effects of tai chi chuan and rTMS. Tai chi chuan, as a mind-body exercise, acts as a widespread priming of the brain,^39^ increasing receptiveness to subsequent inputs. By targeting the DLPFC, low-frequency rTMS can induce distal neural activity while durably modulating neuronal excitability and connectivity, promoting neuroplasticity^40^ and contributing to the amelioration of dysfunctional brain states,^23^ which facilitate brain neural remodeling to improve sleep quality.^41^ Improved nighttime sleep quality enhances the flow of the brain’s glymphatic system, facilitating the clearance of neurotoxic metabolites such as beta-amyloid and tau proteins, which in turn improves daytime cognitive function.^5,42^ The DLPFC has extensive connections with other regions and plays crucial roles in the regulation of cognitive functions.^43^ Disrupting of PFC-related functional circuits has been linked to poor sleep quality,^44^ suggesting that individuals with sleep disorders and MCI may have functional disturbances in PFC network connectivity.^45,46^ Low-frequency rTMS targeting the DLPFC can enhance functional connectivity between the frontal and parietal lobes and can spread to affect distant functional connections within the network.^47^ Furthermore, we combined a neural navigation system with rTMS for precise DLPFC targeting based on individual anatomic locations, enhancing the widespread activation effects of tai chi chuan on the neural plasticity^48^ and thereby improving sleep quality and cognitive function. Moreover, tai chi chuan can modulate the expression of excitatory neurotransmitters,^49,50^ whereas low-frequency rTMS targeting the DLPFC could increase inhibitory neurotransmitter levels,^24,51,52^ thus jointly regulating the balance of excitation and inhibition to alleviate sleep disorders. Further efforts are needed to identify the neural plasticity mechanisms underlying the effects of rTMS combined with tai chi chuan.

Interestingly, practicing tai chi chuan in the early morning is beneficial for burning and using overnight energy reserves, which can increase caloric expenditure and brain neuron activity,^53^ and performing low-frequency rTMS before nighttime sleep can inhibit neural activity, enhancing sleep quality.^47,54^ The combined intervention aligns with the human circadian rhythm, synergistically improving nighttime sleep quality and cognitive function.

### Limitations

This study has several limitations. It lacked wrist actigraphy for objective sleep monitoring during the follow-up phase. Future research should incorporate objective sleep monitoring during follow-up to gain a more accurate understanding of patients’ sleep patterns and long-term changes. Participants were recruited solely from community centers in China, limiting the generalizability of the study. Future research should conduct multicenter clinical trials to include a more diverse range of patient populations. Currently, our clinical efficacy assessment relies mainly on neuropsychologic tests. Future studies should integrate detailed neuroimaging and physiologic assessment methods to reveal the potential mechanisms underlying the effects of these interventions. Our current research has yet to focus on the impact of emerging pharmacologic therapies on sleep disorders. In the future, we will actively assess the effectiveness of combining pharmacologic treatments with rTMS in the management of sleep disorders.

## Conclusions

In this randomized clinical trial, 1-Hz rTMS enhanced the clinical benefits of tai chi chuan by improving sleep quality and cognitive function among older adults with sleep disorders and MCI. The beneficial effects were shown to be long-lasting and stable. These findings provide data on nonpharmacologic strategies for the rehabilitation of sleep disorders that may help delay or prevent MCI. Further studies are needed to assess neural changes associated with this treatment.

## Notes

Supplement 1.
Trial Protocol and Statistical Analysis Plan

Supplement 2. **eTable 1.** Learn the Basic Principles and Moves of Tai Chi Chuan 2 Weeks/14 Days in Advance **eTable 2.** The 6-Week Protocol of Tai Chi Chuan Interventions **eTable 3.** Median (IQR) of Physical Activity of 2 Groups by ITT in Follow-Up **eTable 4.** Hypnotic Medication Usage record **eFigure 1.** The Frequency of Hypnotics Was Changed During the 6-Week Intervention **eTable 5.** Safety Outcomes: Adverse Events **eFigure 2.** Subgroup Analysis of PSQI and MoCA Scores at 12 Weeks by ITT **eFigure 3.** Subgroup Analysis of PSQI and MoCA Scores at 6 Weeks by ITT
eReference

Supplement 3.
Data Sharing Statement
