# Supplementary material for: Repetitive Transcranial Magnetic Stimulation and Tai Chi Chuan for Older Adults With Sleep Disorders and Mild Cognitive Impairment: A Randomized Clinical Trial
Source: JAMA Netw Open. 2025 Jan 10;8(1):e2454307. doi: 10.1001/jamanetworkopen.2024.54307 (PMC12548080; doi:10.1001/jamanetworkopen.2024.54307)
Supplement: Supplement 1. — Trial Protocol and Statistical Analysis Plan [file jamanetwopen-e2454307-s001.pdf]

Protocol and SAP：Repetitive transcranial magnetic stimulation enhances the clinical benefits of Tai Chi Chuan for older adults with sleep disorders and Mild Cognitive Impairment: A Randomized Clinical Trial

Zhizhen Liu, MD, PhD; Lin Zhang, MS; Linxin Bai, MS; Zhenxing Guo, MS; Jiahui Gao, MS; Yong sheng Lin, MS; Yongjin Zhou, PhD, Jinghui Lai, MS, Jing Tao, MD, PhD; Lidian Chen, MD, PhD.

Funding: This study was supported by National Natural Science Foundation of China (Grant No: 82030123). Natural Science Foundation for Distinguished Young Scholars of Fujian Province of China (Grant No: 2022J06028) the scientific research foundation for the top youth talents of Fujian University of Traditional Chinese Medicine (Grant No: XQC2023005)

1、Protocol:

BACKGROUND AND SIGNIFICANCE

Sleep disorders are a significant public health issue worldwide, nearly half of all adults older than 60 years of age report sleep disorders [1,2]. As people age, sleep disorders can accelerate the severity of cognitive decline. Sleep disturbances have been recognized as an independent risk factor for mild cognitive impairment and dementia[3], with a 30% increased risk of dementia [4]. Early improvement in sleep quality is crucial in preventing cognitive decline in the older adults [5,6]. As pharmacological approaches have shown unsatisfactory results [7], the American Geriatrics Society (AGS) recommends prioritizing non-pharmacological strategies to improve sleep issues in the older adults [8].

Lines of evidence showed that exercise is a beneficial strategy to improve sleep quality [9-11]. Tai Chi Chuan, as a mind-body exercise, has been shown to improve sleep disorders in the older adults [12] and delay the progression of cognitive impairment [13], with its benefits closely linked to activation of the prefrontal cortex [14]. However, the functional gains of Tai Chi Chuan remain limited [15], and adhering to a long-term exercise regime is challenging for older adults.

The DLPFC serves as a central regulator of cognitive function [16,17], sleep disorders can disrupt the balance of excitability and inhibitory sham in the brain, including the DLPFC [18], contributing to cognitive decline. Low-frequency rTMS can promote cortical network and subcortical plasticity changes [19] by targeting the DLPFC [20], effectively improving sleep quality [21,22]. Tai Chi Chuan can induce widespread brain plasticity changes [23], when combined with rTMS, can lead to more lasting neural plasticity alterations [24], enhancing brain benefits through exercise, such as significantly improving physical function in patients with Parkinson's disease [25]. We hypothesize that 1-Hz rTMS targeting the right DLPFC could enhance the clinical benefits of Tai Chi Chuan in improving sleep quality and cognitive function in older adults with sleep disorder and MCI, although the efficacy remains unclear.

In this study, we examined whether 1-Hz rTMS stimulation of the right DLPFC was more effective than sham rTMS in augmenting the benefits of Tai Chi Chuan on sleep quality and

cognitive function in older adults with sleep disorder and MCI, using a sham-controlled, double-blind intervention design. We assessed sleep quality and cognitive function at the 6-week postintervention and 12-week follow-up.

Method

Participants

Diagnostic criteria

The diagnostic criteria for sleep disorders are consistent with the diagnostic criteria for primary insomnia in the third edition of the International Classification of Sleep Disorders (ICSD) [26]: 1) Consistent with the diagnostic criteria for primary insomnia in the Diagnostic and Statistical Manual of Mental Disorders (DSM-5) [27]: ① Difficulty initiating and/or maintaining sleep and/or early awakening; ② Clinically significant distress or impairment in important functional areas caused by sleep disorders; ③ Sleep difficulties occurring at least three nights per week; ④ Sleep disorders lasting for at least three months; ⑤ Sleep disorders not affected by sufficient sleep opportunities; ⑥ Subjects without medical/psychiatric disorders, drug abuse, and/or other sleep disorders that cause sleep disorders. 2) Pittsburgh Sleep Quality Index (PSQI) score > 6; 3) No significant history of cognitive impairment or other mental and neurological diseases.

The diagnostic criteria of MCI refer to the 2004 Petersen MCI diagnostic criteria: ①cognitive impairment confirmed by patients or informants, or experienced clinicians; ② one or more cognitive domain impairments (memory, language, visual space, or executive function); ③ basically normal functional activities; and no dementia.

Inclusion Criteria

① Subjects must meet both the diagnostic criteria for sleep disorders and MCI.

② Be aged between 60 and 75;

③ Have not participated in regular physical exercise (at least 3 times a week, with each session lasting at least 20 minutes) in the past three months;

④ Be able to understand and cooperate, willing to participate, and have signed an informed consent form.

Exclusion Criteria

① Patients with a Geriatric Depression Scale-15 (GDS-15) score of ≥9 or who have taken any antipsychotics within the past month;

② Patients with insomnia caused by any severe chronic diseases;

③ Patients with insomnia due to reasons such as shift work or jet lag;

④ Patients who are unable to cooperate in completing Tai Chi Chuan, such as those with motor dysfunction caused by neurological diseases, musculoskeletal system diseases, severe organ diseases, and other contraindications for exercise;

⑤ Patients who are unable to cooperate in completing rTMS treatment, such as those with

central nervous system diseases, including epilepsy, brain tumors, hemangiomas, encephalitis, acute brain injury and other cerebrovascular diseases, as well as those with implanted metal or devices in their bodies;

⑥ Patients who have regularly received TMS, transcranial direct current stimulation, acupuncture, and hypnotic drug therapy within 2 weeks before enrollment;

⑦ Participants who are currently participating in other research trials that may affect the results of this study.

Sample size

Currently, there is no literature on the improvement of sleep disorders through the combination of aerobic exercise and rTMS or Tai Chi Chuan and rTMS. Therefore, the sample size was calculated by referring to the literature on the improvement of PSQI by rTMS and Tai Chi Chuan, and ultimately, the literature with a larger sample size was selected for reference.

According to a study on rTMS treatment for primary insomnia [28], the effect index of sleep quality measured by PSQI scores was 8.02±2.53 in the experimental group and 11.45±6.14 in the control group. With a significance level set at α=0.05 and the test efficiency was 0.80, G Power 3.1 software calculated that each group would require a minimum sample size of 31 participants. Considering a 10% dropout rate, this translates to 335 participants per group, resulting in a total required sample size of 70 participants.

According to the research results of Tai Chi Chuan for the treatment of sleep disorders in elderly women [29] , the effect indicator PSQI score was 11.26±1.52 in the experimental group and 12.48±2.59 in the control group. With the test level α set at 0.05 and the test power at 0.80, the G Power 3.1 software calculated that at least 49 cases were needed in each group. Considering a 10% dropout rate, Therefore, a total of 110 cases were required for this study, 55 cases were needed in each group.

Randomization and Masking

The random grouping sequence of subjects for this study was generated by a specialized statistical staff using the SPSS 24.0 Statistics software program (IBM, Chicago, IL, USA). Employing block randomization (with a block size of 4), the included study participants were randomized in a 1:1 ratio into the experimental group (Tai Chi Chuan + 1-Hz rTMS) and the Sham group (Tai Chi Chuan + Sham-rTMS). Blinding was implemented for outcome assessors and statisticians. The random sequence was managed by a designated individual within the research team who was not involved in subject recruitment, outcome assessment, or statistical analysis. After the trial concludes, data will be entered into the database according to subject codes. Upon verification of data accuracy, the database will be locked, and the first unblinding will be conducted. The individual responsible for the random sequence will provide the grouping codes to the statistician. The second unblinding will occur after completion of data analysis, where the individual managing the random sequence will

reveal the group codes.

Ethics

The study follows the Helsinki Declaration. The research protocol has been reviewed and approved by the Ethics Committee of the Rehabilitation Hospital Affiliated to Fujian University of Traditional Chinese Medicine (Approval No: 2022KY-024-01), and has been registered in the Chinese Clinical Trial Registry (Clinical Registration No: ChiCTR2200063274). All participants voluntarily enrolled in this clinical trial and signed informed consent forms.

Screening

Participants would be recruited from various communities in Fuzhou City, Fu Jian province through posting posters, handing out flyers and pamphlets. Potential participants will complete a brief sleep questionnaire and cognitive screening scale for initial screening to assess their sleep condition and cognitive function, so as to determine whether they meet the research criteria. Then, Potential participants would be invited to participate in a comprehensive face-to-face interview, including the diagnosis of primary insomnia and MCI, as well as a comprehensive questionnaire survey, detailed comorbid medical history and medication use would be obtained, as well as a comprehensive assessment of cognitive function. Before the face-to-face interview, all participants signed an informed consent form. Our clinical collaborators have played a key advisory role in supervising the interviews of this study. If any suspicious cases arise during the recruitment of participants, decisions will be made based on consultation with clinical collaborators.

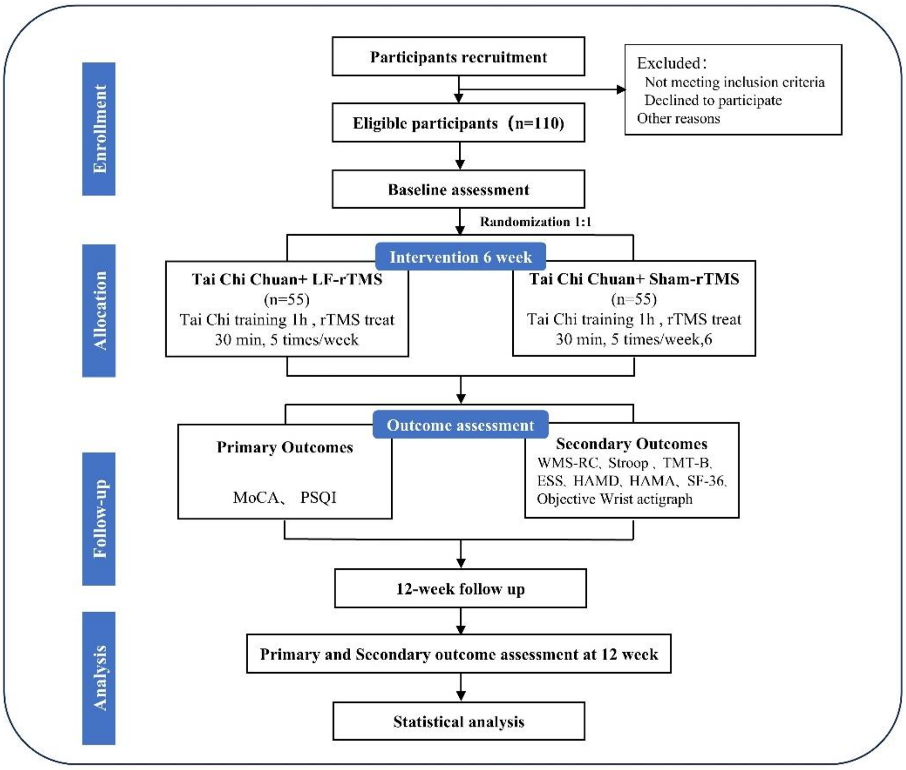

Figure 1. Study flow diagram

Abbreviations: MoCA, Montreal Cognitive Assessment; PSQI, Pittsburgh Sleep Quality Index; WMS-RC, Wechsler memory scale; HAMA, Hamilton Anxiety Rating Scale; HAMD, Hamilton Depression Rating Scale; SF-36, Short Form 36 Health Survey; ESS, Epworth Sleepiness Scale; rTMS, repetitive Transcranial Magnetic Stimulation;

Study Design

This study is a randomized, double-blind, placebo-controlled clinical trial conducted in China between October 2022 and February 2024. Eligible participants will be randomly assigned to two groups: (1) The experimental group (Tai Chi Chuan + 1-Hz rTMS), and (2) The Sham group (Tai Chi Chuan + Sham-rTMS). The flowchart of this trial is shown in **Figure 1**. Assessments will be conducted at the following time points: Screening/Baseline (T1): Conducted two weeks prior to the start of the study/intervention. Intervention (T2): Conducted immediately after the completion of the 6-week intervention. Follow-up (T3): Conducted at 12 weeks after the end of the intervention. At screening, inclusion and exclusion criteria, as well as diagnostic criteria (**Table 1**), will be assessed, and demographic information (such as age, gender, education, medication history in the past two weeks, comorbid, tea, alcohol consumption, and smoking status) will be collected. Cognitive and sleep outcomes will be evaluated at baseline, post-intervention, and follow-up, and objective sleep parameter will be collected using objective wristwatches before and after the intervention. Within 6 weeks after the end of the intervention, the International Physical Activity Questionnaire (IPAQ) will be used to record the exercise status of all participants to monitor the frequency and intensity of exercise throughout the study period.

Table 1 Schedule of assessments

**Visits**	**Study initiation Enrolment/ Baseline T1 week-2-(-1)**	**Intervention[a] T2 week 1-6**	**Follow-up T3 week 12**
Demographics			
Clinical diagnosis of primary insomnia following the DSM-5	X		
Demographics	X		
Comorbid Illnesses and concomitant medications	X		
tea, alcohol intake, smoking status	X		
Geriatric Depression Scale-15	X		
Outcomes			
Montreal cognitive assessment	X	X	X
Pittsburgh Sleep Quality Index	X	X	X
WMS-RC	X	X	X
Stroop test Victoria version	X	X	X

TMT-B	X	X	X
Hamilton Anxiety Rating Scale	X	X	X
Hamilton Depression Rating Scale	X	X	X
Short Form 36 Health Survey	X	X	X
ESS	X	X	X
Wrist actigarphy	X	X	
Safety			
Adverse Events	X	X	X
Other			
International Physical Activity Questionnaire [b]			X

[a] This visit will also take place if the patient withdraws from study or is terminated early
[b] The International Physical Activity Questionnaire will be administered weekly after the intervention

Intervention

Tai Chi Chuan

The Tai Chi Chuan training courses were conducted every morning for 6-week, with 5 sessions per week and a duration of 1 hour per session. The Tai Chi Chuan courses will be instructed by Tai Chi Chuan coaches with at least 5 years of experience. The training process will include a 10-minute warm-up consisting of breathing exercises, relaxation exercises, and standing meditation, followed by a 40-minute standard Tai Chi Chuan training session and a 10-minute cool-down. This study adopts the 24-form/simplified Yang-style Tai Chi training program, which is the most commonly adopted and studied form/style of Tai Chi Chuan in the literature. Similar to regular exercise, Yang-style Tai Chi Chuan is considered moderate-intensity exercise with a metabolic cost of approximately 3.24 METs. During the sessions, the coach will continuously and closely monitor the participants' performance, and can adjust the intensity, range of motion, and number of repetitions when deemed necessary by the coach to ensure safe practice. The researchers will be on-site to oversee the Tai Chi Chuan courses and monitor the fidelity of the treatment protocol in the study. The specific course schedule is detailed in **Supplement 2.**

The complete teaching video of "Yang/24-style Simplified Tai Chi Chuan" refers to the version recommended by the General Administration of Sport of China. The specific link is as follows:https://www.youtube.com/watch?v=_jNG462tlf4&list=PLEV1zx7w0BlCAJOqHLjAwhZySTmI-0RSK

Venue for Tai Chi Chuan and Regular Exercise Classes

The Tai Chi Chuan classes will be conducted in the rest area of the Wen Quan Park in Fuzhou City. The park selected for this study has both open spaces and covered areas. In case of inclement weather, the classes will be moved to the covered areas within the same park. Most

of the classes will be held in the open spaces of the park except for days with bad weather, when they will be conducted within the covered areas of the park. Attendance will be recorded daily, and the compliance of participants who do not attend will be monitored through video recordings and verified by the researchers. It is ensured that the attendance rate for group exercise classes would be above 90%.

1-Hz rTMS

In this study, rTMS intervention was performed using a Magstim Rapid2 stimulator equipped with a standard 70mm figure-of-eight coil (Magstim, Wales, United Kingdom). The rTMS intervention was administered by qualified rehabilitation therapists, starting at 6 pm every evening. Each treatment session lasted for 20 minutes, with 5 sessions per week for a total of 6 weeks. Prior to the treatment, the resting motor threshold (RMT) was measured by applying single-pulse rTMS to the right motor cortex. The RMT was determined as the minimum stimulus intensity required to elicit a clear motor response in the left abductor pollicis brevis (APB) muscle (5 out of 10 trials, with an amplitude of 50 mV). The final stimulus intensity was set at 80% of the resting motor threshold (RMT) for each participant [30].

Each participant's anatomical brain MRI was used for marking of the personal brain regions, determined by projecting the right DLPFC (as determined by anatomical landmarks) onto the scalp. Using the system's integrated neuronavigation unit, continuous monitoring of the precise TMS location relative to the right DLPFC was performed. Daily sessions applied across right DLPFC for a total of 1200 pulses with train duration of 1 second using 1-Hz rTMS During the entire stimulation process， over a continuous duration of 20 minutes, all participants sat in a chair with the coil placed tangentially at a 45° angle to the scalp, with the handle pointing backward towards the midline. Participants were instructed to sit in a semi-reclining chair, remain relaxed, and wear earplugs to prevent noise interference. The rTMS intervention was administered by a qualified rehabilitation therapist.

Sham-rTMS

The sham stimulation group received the same rTMS intervention. The sham rTMS coil (sham air film coil, Magstim, Wales, UK) has the same symmetrical mechanical design as the active coil, and is identical in overall shape or appearance to the real rTMS coil, but does not produce active stimulation to the underlying cortical tissue. It was also placed on the right DLPFC of the participants, and the sham stimulation group received rTMS treatment with the same parameters as the real stimulation group. Compliance (rTMS intensity) will be measured and recorded.

Outcomes

The outcome indicators of this study will be evaluated at baseline assessment (before intervention), 6-week after intervention, and at the 12-week follow-up. Each patient

underwent a series of neuropsychological assessments to evaluate their cognitive level and sleep status. The examinations were conducted in accordance with a unified implementation plan and standard operating procedures.

Primary Outcomes

Sleep quality assessment: Use the internationally recognized Pittsburgh Sleep Quality Index (PSQI) to assess the sleep quality of the subjects in the past month. The PSQI scale consists of 19 self-assessment items, comprising 7 dimensions: sleep quality, sleep onset latency, sleep duration, sleep efficiency, sleep disturbances, use of sleep medication, and daytime dysfunction. Each dimension is scored on a scale of 0-3, with the cumulative score of all dimensions representing the total PSQI score, which ranges from 0 to 21. A higher score indicates poorer sleep quality.

Global cognitive function: The Montreal Cognitive Assessment (MoCA) (FuZhou Edition) was used to assess the Global cognitive function of patients. which includes 8 cognitive domains and 11 examination items covering visuospatial and executive functions, naming, memory, attention, language, abstraction, delayed recall, and orientation. The MoCA score ranges from 0 to 30, with higher scores indicating better overall cognitive function. If the education level is ≤12 years, an additional point is added to the total score, and a score of <26 indicates mild cognitive impairment (MCI).

Secondary outcomes

Memory function assessment:

Wechsler Memory Scale (WMS-RC): Widely used for assessing memory function, the WMS-RC reflects the overall level of memory function. WMS-R mainly tests the functions of immediate memory, working memory, and long-term memory. The final score of WMS-RC is calculated as MQ, and a higher MQ score indicates better memory.

Executive function assessment:

Stroop test Victoria version: The classic Stroop test includes interference and control tasks. The Victoria Stroop Test (VST) uses three conditions, including naming the color of dots, the color of neutral words, and the color of colored words printed in incongruent colors. Participants are required to quickly identify the color of a series of words. The tester records the "total time taken".

Attention

Trial Making Test Part B (TMT-B): TMT-B consists of numbers 1-25 in circles and squares, requiring participants to connect the numbers in ascending order as quickly as possible while alternating between the two shapes. The evaluator records the time taken to complete the task, with shorter completion times indicating better performance.

The objective sleep quality: The Wrist actigraphy, which integrates a three-axis accelerometer and an optical heart rate sensor, was used to record objective sleep quality data

[31]. The subjects wore the device on their non-dominant wrist before going to bed. The built-in three-axis accelerometer recorded the acceleration signals of the wrist and converted them into digital signals for storage. After connecting the finger cuff with the optical heart rate sensor to the body movement recorder, the photoelectric pulse wave recording method was used to collect pulse wave signals by emitting infrared light to the skin. The sleep status of the subjects was monitored for seven consecutive nights at baseline and after treatment. Objective sleep data would be extracted after the participants returned the wrist-worn body movement recorder, and subsequent data analysis would be conducted. This included sleep efficiency (total sleep time / total time in bed x 100%), time of arousal after falling asleep, number of arousals per night, sleep latency, total sleep time, and average arousal time per arousal.

Epworth Sleepiness Scale (ESS): This scale contains eight scenarios describing daily life situations. Subjects are required to evaluate their tendency towards sleepiness based on these scenarios. Each scenario has a score ranging from 0 to 3, indicating the degree of sleepiness tendency in the corresponding situation. The total score ranges from 0 to 24. A score greater than 6 suggests drowsiness, greater than 11 indicates excessive drowsiness, and greater than 16 suggests dangerous drowsiness.

Hamilton Anxiety Rating Scale (HAMA): This scale is used to assess patients' anxiety symptoms over the past week, including anxiety level, tension, fear, and other manifestations. HAMA consists of 14 items, with scores ranging from 0 to 4, to assess the severity of anxiety symptoms. The higher the total score, the more severe the patient's anxiety symptoms.

Hamilton Depression Rating Scale (HAMD): This scale is used to assess patients' depressive symptoms over the past week, including moodiness, insomnia, self-reproach, helplessness, and other manifestations. HAMD consists of 17 items, with scores ranging from 0 to 4, to assess the severity of depressive symptoms. The higher the total score, the more severe the patient's depressive symptoms.

The Short Form 36 Health Survey (SF-36): SF-36 was used to assess changes in the subjects' quality of life. A higher score indicates better quality of life.

Data management

During assessments, assessors will complete the CRF according to the working manual. All CRFs will be dated and signed by the responsible researcher or an authorized staff member responsible for the quality and accuracy of the data collection process. Subsequently, two research assistants will enter the raw data into an Excel spreadsheet using double data entry. In case of inconsistencies during the data entry process, a third party may be consulted to ensure data accuracy.

2、Statistical Analysis Plan

Data set

After the electronic database was locked, the database was divided into Intention-to-Treat analysis (ITT), Per Protocol analysis (PP), and Safety Set (SS) using the following criteria.
Intention-to-treat (ITT) refers to all participants who were randomized into different treatment groups, regardless of whether they completed the treatment as assigned or not.
Per protocol (PP) refers to the set of cases that meet the inclusion criteria, do not meet the exclusion criteria, and have completed the treatment protocol. Each subject in the PP dataset is a valid case or sample with good adherence, no protocol violations, and complete baseline values for key indicators. Prespecified sensitivity analyses were conducted using complete-case data.
Safety set (SS) refers to the actual data for subjects who receive at least one intervention after randomization and for whom safety indicators are documented. The incidence of adverse reactions is calculated using the number of cases in the safety set.
Adverse events: Will be analyzed, with the number of cases, category, and severity of occurrences counted separately and their relationship to the intervention.

Covariates and subgroup analysis

If there are statistically significant differences in the inter-group comparison of baseline indicators, covariates should be included in the analysis after intervention for correction. Use Generalized Estimating Equation (GEE) model to analyze primary and secondary outcome measurements. While including baseline measurements and IPAQ as covariates in the analysis. In addition, subgroup analysis will be performed by sex, age, education, BMI at baseline**,** GDS-15. The subgroup analysis will be carried out as exploratory.

Missing data

For missing data in this study， the percentage of missing data will be reported, the potential patterns of missing data should be examined, and appropriate method should be used for multiple imputation of missing data. The multiple imputation method will be preferred for analyzing the missing data, and the complete-case data should be reported in the manuscript as sensitivity analysis. The patients' demographic characteristics were involved in the missing data model for multiple imputation, and the number of multiple imputation will be set as 5.

Data management and general analysis

A comparison of baseline characteristics of the selected subjects in the two groups was conducted to assess comparability. Intervention effect evaluation included determining

intervention effect indicators and comparing intervention effects between groups. The primary outcome evaluation used the ITT dataset as the main dataset for intervention effect evaluation and the PP dataset for sensitivity analysis. Secondary outcome indicators were evaluated using the PP dataset. Safety assessment included statistical analysis of adverse events such as dizziness and falls, with the SS dataset used for safety assessment.

All statistical analyses were performed using SPSS 24.0 software. Two-tailed tests were used for statistical analysis, the primary outcome indicator is $P<0.025$ and the secondary outcome indicator is $P<0.05$ are considered statistically significant in comparison between groups. Continuous data were presented as means, standard deviations, or medians, and interquartile ranges depending on the normality of distribution. Categorical data were presented as frequencies (percentages). Normality tests were conducted for variable distributions, and independent sample t-tests were used for intergroup comparisons of normally distributed continuous data. Non-parametric tests were used for statistical analysis of non-normally distributed continuous data. Chi-square tests were used for comparisons of categorical variables, with the Fisher exact probability test used when necessary.

Analysis of primary endpoint

Statistical analysis of the primary outcome indicators was conducted using the ITT dataset and the PP dataset. The baseline measurements of the study subjects were adjusted using GEE model to compare the intervention effects in each group. At a single time point, the intergroup differences at baseline, 6-week postintervention, and during 12-week follow-up are compared using independent sample t-tests or Mann-Whitney U tests.

Analysis of secondary endpoints

Statistical analysis of the secondary outcome indicators was performed using the PP dataset. The baseline measurements of the study subjects were adjusted using a generalized estimating equation model to compare the intervention effects in each group. At a single time point, the intergroup differences at baseline, 6-week postintervention, and during 12-week follow-up are compared using independent sample t-tests or Mann-Whitney U tests.

Safety

During the study, any accidental injuries and acute illnesses will be recorded as Adverse Events (AEs). AEs will be classified as mild, moderate, or severe. Serious Adverse Events (SAEs) are events that result in life-threatening conditions, require hospitalization, or lead to persistent severe disabilities. All AEs and SAEs that are accidental or potentially related to the study will be reported to the Ethics Committee. The researchers will document the severity of symptoms, occurrence time, duration, and treatment measures in the Case Report Form (CRF) and assess their relevance to the intervention training.

3、Quality Control

Quality Control for Researchers

Before the start of the project, a detailed research manual will be developed and all researchers will undergo centralized and unified training to ensure the quality of the project's completion. This training will ensure that recruitment personnel are familiar with the recruitment process, screening, and inclusion/exclusion criteria; assessment personnel are proficient in the standardized assessment and calculation rules for outcome measures; objective sleep quality data is collected in a standardized manner; supervisory personnel are on-site to oversee training sessions and record participant attendance; and data entry personnel perform double entry and verification of data. Each step of the process will be executed by designated personnel who will not participate in other aspects of the study.

Quality Control for Participants

All participants will be strictly screened based on the inclusion and exclusion criteria, and those who do not meet the criteria will be excluded, with the number of exclusions and reasons for exclusion recorded. Prior to the start of the study, each participant will be provided with a detailed explanation of all experimental tasks and intervention methods, to address any concerns and provide free sleep, cognitive testing, and health consultations. Participant informed consent will be obtained.

Quality Control for Intervention Processes

During the Tai Chi training sessions, professional coaches will provide on-site teaching and guidance, promptly correcting any improper movements. Researchers will oversee each training session in its entirety, implementing an on-site sign-in system to track attendance. Participants who cannot attend scheduled training sessions on time are required to submit advance leave requests, with reasons for leave recorded. Participants who temporarily cannot attend centralized training sessions are instructed to practice at home using the day's teaching video and submit recorded practice videos to the Tai Chi coach for feedback. Tai Chi training sessions will be conducted in appropriate venues based on weather conditions to enhance participant compliance.

During rTMS treatment, the operation of the rTMS device must be performed by qualified rehabilitation therapists, the researchers promptly observe the participant's response and record the treatment intensity, frequency, and adverse reactions. Additionally, during the treatment, measures were taken to ensure that patients could not differentiate between the real and sham coils based on appearance, sound, or the sensation of stimulation, thereby maintaining blinding for the patients. Due to the specific settings and adjustments required for the equipment during operation, the therapists were aware of whether they were using an active coil or a sham coil while delivering stimulation. This was done to ensure the smooth conduct of the experiment. However, for the evaluators, we ensured that they were completely

unaware of the status of the coils, as their sole responsibility was to collect and analyze the data.

Quality Control for Data Collection

When using wrist-worn activity monitors to objectively assess sleep, participants will be instructed to wear the monitor correctly on their wrist, ensuring a close fit to the skin to prevent loosening or falling off during sleep. Before each monitoring session, the monitor will be calibrated and tested to confirm its proper functioning, ensuring the accuracy and reliability of data monitoring.

Reference：

1. Li J, Vitiello MV, Gooneratne NS. Sleep in Normal Aging. *Sleep medicine clinics.* 2022;17(2):161-171.
2. Ma GY, Cai L, Fan LM, et al. Association of socioeconomic factors and prevalence of hypertension with sleep disorder among the elderly in rural southwest China. *Sleep medicine.* 2020;71:106-110.
3. Nedelec T, Couvy-Duchesne B, Monnet F, et al. Identifying health conditions associated with Alzheimer's disease up to 15 years before diagnosis: an agnostic study of French and British health records. *The Lancet Digital health.* 2022;4(3):e169-e178.
4. Sabia S, Fayosse A, Dumurgier J, et al. Association of sleep duration in middle and old age with incidence of dementia. *Nature communications.* 2021;12(1):2289.
5. Yaffe K, Falvey CM, Hoang T. Connections between sleep and cognition in older adults. *The Lancet Neurology.* 2014;13(10):1017-1028.
6. Wang Q, Xu S, Liu F, et al. Causal relationship between sleep traits and cognitive impairment: A Mendelian randomization study. *Journal of evidence-based medicine.* 2023;16(4):485-494.
7. Riemann D, Nissen C, Palagini L, Otte A, Perlis ML, Spiegelhalder K. The neurobiology, investigation, and treatment of chronic insomnia. *The Lancet Neurology.* 2015;14(5):547-558.
8. American Geriatrics Society 2019 Updated AGS Beers Criteria® for Potentially Inappropriate Medication Use in Older Adults. *Journal of the American Geriatrics Society.* 2019;67(4):674-694.
9. Kelley GA, Kelley KS. Exercise and sleep: a systematic review of previous meta-analyses. *Journal of evidence-based medicine.* 2017;10(1):26-36.
10. Jurado-Fasoli L, De-la OA, Molina-Hidalgo C, Migueles JH, Castillo MJ, Amaro-Gahete FJ. Exercise training improves sleep quality: A randomized controlled trial. *European journal of clinical investigation.* 2020;50(3):e13202.
11. Ferreira MF, Bos SC, Macedo AF. The impact of physical activity on objective sleep of people with insomnia. *Psychiatry research.* 2023;320:115019.
12. Siu PM, Yu AP, Tam BT, et al. Effects of Tai Chi or Exercise on Sleep in Older Adults With Insomnia: A Randomized Clinical Trial. *JAMA network open.* 2021;4(2):e2037199.
13. Chen Y, Qin J, Tao L, et al. Effects of Tai Chi Chuan on Cognitive Function in Adults 60 Years or Older With Type 2 Diabetes and Mild Cognitive Impairment in China: A Randomized Clinical Trial. *JAMA network open.* 2023;6(4):e237004.
14. Wu MT, Tang PF, Goh JOS, et al. Task-Switching Performance Improvements After Tai Chi Chuan Training Are Associated With Greater Prefrontal Activation in Older Adults. *Frontiers in aging neuroscience.* 2018;10:280.

15. Wei L, Chai Q, Chen J, et al. The impact of Tai Chi on cognitive rehabilitation of elder adults with mild cognitive impairment: a systematic review and meta-analysis. *Disability and rehabilitation.* 2022;44(11):2197-2206.
16. Li X, Qi G, Yu C, et al. Cortical plasticity is correlated with cognitive improvement in Alzheimer's disease patients after rTMS treatment. *Brain stimulation.* 2021;14(3):503-510.
17. Ma S, Skarica M, Li Q, et al. Molecular and cellular evolution of the primate dorsolateral prefrontal cortex. *Science (New York, NY).* 2022;377(6614):eabo7257.
18. Lanza G, Cantone M, Lanuzza B, et al. Distinctive patterns of cortical excitability to transcranial magnetic stimulation in obstructive sleep apnea syndrome, restless legs syndrome, insomnia, and sleep deprivation. *Sleep medicine reviews.* 2015;19:39-50.
19. Fitzsimmons S, Oostra E, Postma TS, van der Werf YD, van den Heuvel OA. Repetitive Transcranial Magnetic Stimulation-Induced Neuroplasticity and the Treatment of Psychiatric Disorders: State of the Evidence and Future Opportunities. *Biological psychiatry.* 2024;95(6):592-600.
20. Eshel N, Keller CJ, Wu W, et al. Global connectivity and local excitability changes underlie antidepressant effects of repetitive transcranial magnetic stimulation. *Neuropsychopharmacology : official publication of the American College of Neuropsychopharmacology.* 2020;45(6):1018-1025.
21. Lanza G, Fisicaro F, Cantone M, et al. Repetitive transcranial magnetic stimulation in primary sleep disorders. *Sleep medicine reviews.* 2023;67:101735.
22. Feng J, Zhang Q, Zhang C, Wen Z, Zhou X. The Effect of sequential bilateral low-frequency rTMS over dorsolateral prefrontal cortex on serum level of BDNF and GABA in patients with primary insomnia. *Brain and behavior.* 2019;9(2):e01206.
23. Jasim N, Balakirishnan D, Zhang H, Steiner-Lim GZ, Karamacoska D, Yang GY. Effects and mechanisms of Tai Chi on mild cognitive impairment and early-stage dementia: a scoping review. *Systematic reviews.* 2023;12(1):200.
24. Hendrikse J, Kandola A, Coxon J, Rogasch N, Yücel M. Combining aerobic exercise and repetitive transcranial magnetic stimulation to improve brain function in health and disease. *Neuroscience and biobehavioral reviews.* 2017;83:11-20.
25. Chung CL, Mak MK, Hallett M. Transcranial Magnetic Stimulation Promotes Gait Training in Parkinson Disease. *Annals of neurology.* 2020;88(5):933-945.
26. Sateia MJ. International classification of sleep disorders-third edition: highlights and modifications. *Chest.* 2014;146(5):1387-1394.
27. Regier DA, Kuhl EA, Kupfer DJ. The DSM-5: Classification and criteria changes. *World psychiatry : official journal of the World Psychiatric Association (WPA).* 2013;12(2):92-98.
28. Jiang CG, Zhang T, Yue FG, Yi ML, Gao D. Efficacy of repetitive transcranial magnetic stimulation in the treatment of patients with chronic primary insomnia. *Cell biochemistry and biophysics.* 2013;67(1):169-173.
29. Song J, Wei L, Cheng K, et al. The Effect of Modified Tai Chi Exercises on the Physical Function and Quality of Life in Elderly Women With Knee Osteoarthritis. *Frontiers in aging neuroscience.* 2022;14:860762.
30. He Y, Li Z, Cao L, et al. Effects of dorsolateral prefrontal cortex stimulation on network topological attributes in young individuals with high-level perceived stress: A randomized controlled trial. *Psychiatry research.* 2023;326:115297.
31. Liu Z, Zhang L, Wu J, et al. Machine learning-based classification of circadian rhythm

characteristics for mild cognitive impairment in the elderly. *Frontiers in public health.* 2022;10:1036886.
