# Supplementary material for: Repetitive Transcranial Magnetic Stimulation and Tai Chi Chuan for Older Adults With Sleep Disorders and Mild Cognitive Impairment: A Randomized Clinical Trial
Source: JAMA Netw Open. 2025 Jan 10;8(1):e2454307. doi: 10.1001/jamanetworkopen.2024.54307 (PMC12548080; doi:10.1001/jamanetworkopen.2024.54307)
Supplement: Supplement 2. — eTable 1. Learn the Basic Principles and Moves of Tai Chi Chuan 2 Weeks/14 Days in Advance eTable 2. The 6-Week Protocol of Tai Chi Chuan Interventions eTable 3. Median (IQR) of Physical Activity of 2 Groups by ITT in Follow-Up eTable 4. Hypnotic Medication Usage record eFigure 1. The Frequency of Hypnotics Was Changed During the 6-Week Intervention eTable 5. Safety Outcomes: Adverse Events eFigure 2. Subgroup Analysis of PSQI and MoCA Scores at 12 Weeks by ITT eFigure 3. Subgroup Analysis of PSQI and MoCA Scores at 6 Weeks by ITT eReference [file jamanetwopen-e2454307-s002.pdf]

Supplemental Online Content

Liu Z, Zhang L, Bai L, et al. Repetitive transcranial magnetic stimulation and tai chi chuan for older adults with sleep disorders and mild cognitive impairment: a randomized clinical trial. *JAMA Netw Open*. 2025; 8(1):e2454307. doi:10.1001/jamanetworkopen.2024.54307

eTable 1. Learn the Basic Principles and Moves of Tai Chi Chuan 2 Weeks/14 Days in Advance
eTable 2. The 6-Week Protocol of Tai Chi Chuan Interventions
eTable 3. Median (IQR) of Physical Activity of 2 Groups by ITT in Follow-Up
eTable 4. Hypnotic Medication Usage record
eFigure 1. The Frequency of Hypnotics Was Changed During the 6-Week Intervention
eTable 5. Safety Outcomes: Adverse Events
eFigure 2. Subgroup Analysis of PSQI and MoCA Scores at 12 Weeks by ITT
eFigure 3. Subgroup Analysis of PSQI and MoCA Scores at 6 Weeks by ITT
eReference

This supplemental material has been provided by the authors to give readers additional information about their work.

© 2025 Liu Z et al. *JAMA Network Open*.

eTable 1. Learn the Basic Principles and Moves of Tai Chi Chuan 2 Weeks/14 Days in Advance

Session	Tai Chi intervention (prepare)	Session	Tai Chi intervention (prepare)
1	• Introduction of Tai Chi, including the history, theory and basic working principle.<br>• 15 min breathing exercise、relaxation exercise<br>• 20 min standing pose meditation<br>• 20 min basic technique of weight shifting and walking	8	• 10 min warm-up exercise<br>• 20 min practice previously learnt forms<br>• 20 min learn “high pat on horse”<br>• 20 min learn “right heel kick”<br>• 10 min breathing and relaxation exercise
2	• 10 min warm-up exercise<br>• 20 min basic technique of weight shifting and walking<br>• 20 min learn “starting pose of the 24 forms”<br>• 10 min breathing and relaxation exercise	9	• 10 min warm-up exercise<br>• 40 min practice previously learnt forms<br>• 10 min breathing and relaxation exercise
3	• 10 min warm-up exercise<br>• 20 min learn and practice “starting pose of the 24 forms”<br>• 20 min learn “part the wild horse's mane”<br>• 20 min learn “white crane spreads its wings”<br>• 10 min breathing and relaxation exercise	10	• 10 min warm-up exercise<br>• 20 min practice previously learnt forms<br>• 20 min learn “strike to ears with both fists”<br>• 20 min learn “turn body and left heel kick”<br>• 10 min breathing and relaxation exercise
4	• 10 min warm-up exercise<br>• 40 min practice previously learnt forms<br>• 10 min breathing and relaxation exercise	11	• 10 min warm-up exercise<br>• 20 min practice previously learnt forms<br>• 20 min learn “left lower body and stand on one leg” and “right lower body and stand on one leg”<br>• 20 min learn “shuttle back and forth”<br>•10 min breathing and relaxation exercise
5	• 10 min warm-up exercise<br>• 20 min practice previously learnt forms<br>• 20 min learn “brush knee and twist step”<br>• 20 min learn “playing the lute”<br>• 10 min breathing and relaxation exercise	12	• 10 min warm-up exercise<br>• 20 min practice previously learnt forms<br>• 20 min learn “needle at sea bottom”<br>• 20 learn “fan through back”<br>•10 min breathing and relaxation exercise
6	• 10 min warm-up exercise<br>• 20 min practice previously learnt forms<br>•20 min learn “reverse reeling forearm”<br>• 20 min learn “grasp sparrow's tail”<br>• 10 min breathing and relaxation exercise	13	• 10 min warm-up exercise<br>• 20 min practice previously learnt forms<br>• 20 min learn “turn body, deflect, parry, and punch”<br>• 20 min learn “appears closed”, “cross hands” and “closing”<br>•10 min breathing and relaxation exercise

© 2025 Liu Z et al. *JAMA Network Open*.

7	• 10 min warm-up exercise<br>• 20 min practice previously learnt forms<br>• 20 min learn “single whip”<br>• 20 min learn “wave hands like clouds”<br>• 10 min breathing and relaxation exercise	14	• 10 min warm-up exercise<br>• 40 min practice previously learnt forms with refinement of posture, movement and meditation components<br>• 10 min breathing and relaxation exercise

Two weeks before the formal intervention was the learning phase. At this stage, the instructor demonstrated Tai Chi moves, breaking each move down into its individual components. Participants mimicked and practiced the movements during coaching and correction. At the end of this session, each participant was asked to complete the entire exercise set independently with music playing in the background.

© 2025 Liu Z et al. *JAMA Network Open*.

eTable 2. The 6-Week Protocol of Tai Chi Chuan Interventions

Session	Tai Chi Chuan intervention	Session	Tai Chi Chuan intervention
1-week	• 10 min warm-up exercise<br>• 40 min practice previously learnt forms with refinement of posture, movement and meditation components<br>• 10 min breathing and relaxation exercise	4-week	• 10 min warm-up exercise<br>• 40 min practice previously learnt forms with refinement of posture, movement and meditation components<br>• 10 min breathing and relaxation exercise
2-week	• 10 min warm-up exercise<br>• 40 min practice previously learnt forms with refinement of posture, movement and meditation components<br>• 10 min breathing and relaxation exercise	5-week	• 10 min warm-up exercise<br>• 40 min practice previously learnt forms with refinement of posture, movement and meditation components<br>• 10 min breathing and relaxation exercise
3-week	• 10 min warm-up exercise<br>• 40 min practice previously learnt forms with refinement of posture, movement and meditation components<br>• 10 min breathing and relaxation exercise	6-week	• 10 min warm-up exercise<br>• 40 min practice previously learnt forms with refinement of posture, movement and meditation components<br>• 10 min breathing and relaxation exercise

eTable 3. Median (IQR) of Physical Activity of 2 Groups by ITT in Follow-Up

ITT	Median (IQR)	
	Experimental n=55	Sham n=55
Total METs[a] (minwk^{-1})	2190(1680)	1908(2133)
Vigorous METs (minwk^{-1})	0(0)	0(0)
Moderate METs (minwk^{-1})	840(1020)	720(1200)
Walking METs (minwk^{-1})	1386(594)	1386(693)
Sedentary time (min)	240(270)	300(240)

[a] METs=Amount of metabolic equivalent minutes per week (METs min·wk^{-1})in the IPAQ
[b] Vigorous intensity =8.0 METs
[c] Moderate intensity =4.0 METs
[d] Walking intensity =3.3 METs

© 2025 Liu Z et al. *JAMA Network Open*.

eTable 4. Hypnotic Medication Usage Record

Hypnotic medication usage	Experimental n = 7	Sham n = 5	*P* value
Intervention 0-3 week	3.4 (1.7)	3.0 (2.4)	.43
Intervention 4-6 week	2.1 (1.9)	2.60 (2.1)	.76

eFigure 1. The Frequency of Hypnotics Was Changed During the 6-Week Intervention

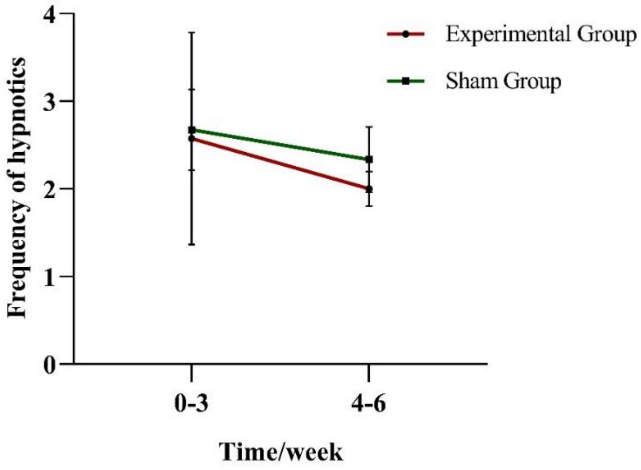

© 2025 Liu Z et al. *JAMA Network Open*.

eTable 5. Safety Outcomes: Adverse Events

Adverse Events	Experimental n = 53	Sham n = 54	*P* value [c]
Total	2	5	.45
Dizzy	0	2	
Fall	1	1	
Hospitalizations	1	1	
Emergency department visits	0	1	

[c] Using the corrected chi-square test, the adjusted χ^2 =0.572.

Subgroup Analysis

Subgroup analysis at 12 weeks revealed that the experimental group had lower PSQI scores than did the sham group, particularly for female (mean difference, -2.29 [95% CI, -3.88 to -0.69]), individuals with more than 10 years of education (mean difference, -2.49 [95% CI, -3.95 to -1.04]), individuals aged 67 years and younger (mean difference, -2.72 [95% CI, -4.3 to -1.15]), participants with a BMI ≤ 24 (mean difference, -2.38 [95% CI, -3.83 to -0.94]), and participants with a GDS-15 score>4 (mean difference, -4.83 [95% CI, -7.15 to -2.51]).

Subgroup analysis at 12-week also demonstrated that compared to the sham group, the experimental group showed an improvement in MoCA scores, especially among female (mean difference, 1.71 [95% CI, 0.57 to 2.85]), individuals older than 67 years (mean difference, 1.4 [95% CI, 0.16 to 2.64]), those with a BMI ≤ 24 (mean difference, 1.71 [95% CI, 0.57 to 2.87]), and participants with a GDS-15 score ≤4 (mean difference, 1.27 [95% CI, 0.35 to 2.2]) (eFigure 2). Subgroup analysis at 6-week showed significant improvements in both PSQI and MoCA scores in all populations undergoing 1-Hz rTMS and Tai Chi Chuan (eFigure 3).

eFigure 2. Subgroup Analysis of PSQI and MoCA Scores at 12 Weeks by ITT

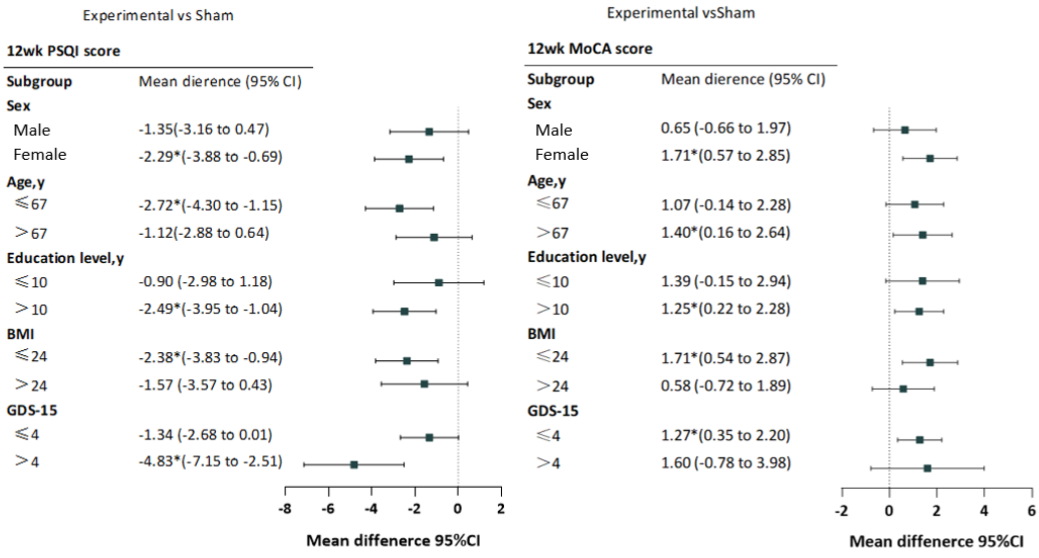

© 2025 Liu Z et al. *JAMA Network Open*.

eFigure 3. Subgroup Analysis of PSQI and MoCA Scores at 6 Weeks by ITT

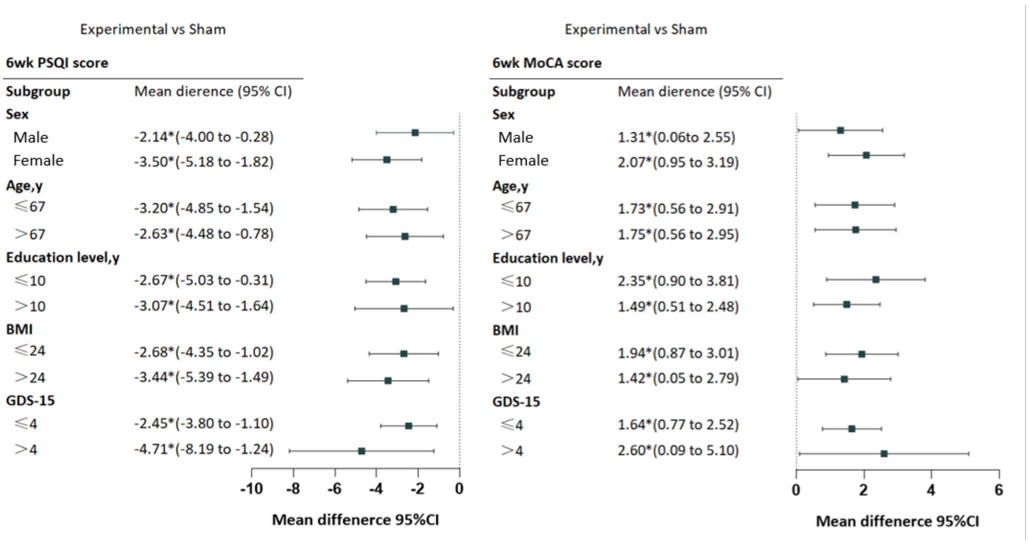

Abbreviations: Pittsburgh Sleep Quality Index (PSQI), Montreal Cognitive Assessment (MoCA), Intention-to-Treat (ITT) Analysis, BMI indicates body mass index (calculated as weight in kilograms divided by height in meters squared), Geriatric Depression Scale-15, GDS-15.

In addition, the subgroup analysis of this study showed that in the experimental group, older adults who were female, had higher education levels, and were not overweight experienced greater improvements in sleep quality and cognitive function. Additionally, older adults with negative emotions may experience greater improvements in sleep quality. This indicates that a combined intervention for improving sleep also alleviates negative emotions. This may be related to the association between rapid eye movement sleep and the decoupling of dendrites in ventral PFC pyramidal neurons, which optimize emotional responses to behavioral stressors [1].

eReference

1. Aime M, Calcini N, Borsa M, et al. Paradoxical somatodendritic decoupling supports cortical plasticity during REM sleep. *Science (New York, NY)*. 2022;376(6594):724-730.

© 2025 Liu Z et al. *JAMA Network Open*.
