# Supplementary material for: Repetitive Transcranial Magnetic Stimulation and Tai Chi Chuan for Older Adults With Sleep Disorders and Mild Cognitive Impairment: A Randomized Clinical Trial
Source: JAMA Netw Open. 2025 Jan 10;8(1):e2454307. doi: 10.1001/jamanetworkopen.2024.54307 (PMC12548080; doi:10.1001/jamanetworkopen.2024.54307)
Supplement: Supplement 3. — Data Sharing Statement [file jamanetwopen-e2454307-s003.pdf]

Data Sharing Statement

Liu. Repetitive Transcranial Magnetic Stimulation and Tai Chi Chuan for Older Adults With Sleep Disorders and Mild Cognitive Impairment. *JAMA Netw Open*. Published January 10, 2025. doi:10.1001/jamanetworkopen.2024.54307

Data
Additional Information: Clinical Trials. gov Identifier: ChiCTR2200063274
Data available: Yes
Data types: Deidentified participant data
How to access data: Contact corresponding author: cld@fjtcm.edu.cn
When available: With publication

Supporting Documents
Document types: None

Additional Information
Who can access the data: Researchers whose proposed use of the data has been approved
Types of analyses: Academic purpose
Mechanisms of data availability: with investigator support
